# Frizzled 7 Expression Is Positively Regulated by SIRT1 and β-Catenin in Breast Cancer Cells

**DOI:** 10.1371/journal.pone.0098861

**Published:** 2014-06-04

**Authors:** Glenn E. Simmons, Somnath Pandey, Ana Nedeljkovic-Kurepa, Madhurima Saxena, Allison Wang, Kevin Pruitt

**Affiliations:** 1 Department of Molecular and Cellular Physiology, LSU Health Sciences Center School of Medicine in Shreveport, Shreveport, Louisiana, United States of America; 2 The Feist-Weiller Cancer Center, LSU Health Sciences Center School of Medicine in Shreveport, Shreveport, Louisiana, United States of America; University of Alabama at Birmingham, United States of America

## Abstract

The Wnt signaling pathway is often chronically activated in diverse human tumors, and the Frizzled (FZD) family of receptors for Wnt ligands, are central to propagating oncogenic signals in a β-catenin-dependent and independent manner. SIRT1 is a class III histone deacetylase (HDAC) that deacetylates histone and non-histone proteins to regulate gene transcription and protein function. We previously demonstrated that SIRT1 loss of function led to a significant decrease in the levels of Dishevelled (Dvl) proteins. To further explore this connection between the sirtuins and components of the Wnt pathway, we analyzed sirtuin-mediated regulation of FZD proteins. Here we explore the contribution of sirtuin deacetylases in promoting constitutive Wnt pathway activation in breast cancer cells. We demonstrate that the use of small molecule inhibitors of SIRT1 and SIRT2, and siRNA specific to SIRT1, all reduce the levels of FZD7 mRNA. We further demonstrate that pharmacologic inhibition of SIRT1/2 causes a marked reduction in FZD7 protein levels. Additionally, we show that β-catenin and c-Jun occupy the 7 kb region upstream of the transcription start site of the FZD7 gene, and SIRT1 inhibition leads to a reduction in the occupancy of both β-catenin and c-Jun at points along this region. This work uncovers a new mechanism for the regulation of FZD7 and provides a critical new link between the sirtuins and FZD7, one of the earliest nodal points from which oncogenic Wnt signaling emanates. This study shows that inhibition of specific sirtuins may provide a unique strategy for inhibiting the constitutively active Wnt pathway at the level of the receptor.

## Introduction

Sirtuin-1 (SIRT1) is an NAD^+^-dependent deacetylase that enables cells to cope with diverse physiological stresses by deacetylating transcription factors, histones, coactivators, enzymes and chromatin regulators to promote cell survival. This diversity in the proteins that it targets for deacetylation, particularly under conditions of cellular stress, may explain why SIRT1 is upregulated in a number of human tumors. For example, several reports have demonstrated SIRT1 upregulation in human cancers including, invasive human ductal carcinoma [1], malignant human breast carcinoma [2], hepatocellular carcinoma [3], diffuse B-cell lymphoma [4], gastric carcinoma [5], and colorectal cancer with microsatellite instability and CpG island methylator phenotype [6]. Additionally, studies involving the influence of SIRT1 deficiency on *in vivo* tumorigenesis have shown that SIRT1 deficiency confined to the intestines led to reduced polyp and tumor formation. APC+/min mice bearing enterocyte-specific inactivation of SIRT1 showed that SIRT1-inactivation reduced the total number and surface of polyps and tumors. Moreover, tumors in SIRT1-deficient mice exhibited markedly increased numbers of cells undergoing apoptosis [7].

While some mouse models have suggested that SIRT1 may promote genetic stability and suppress context-dependent tumorigenesis [8], the oncogenic contribution of SIRT1 has been demonstrated in diverse contexts. For example, SIRT1 has been shown to participate in silencing tumor suppressor genes [9], [10], stabilization of Dishevelled and β-catenin [11], promotion of cell migration [11]–[13], aromatase expression [1], estrogen receptor signaling [14] and chemoresistance to conventional chemotherapeutic agents [15], [16]

One interesting aspect of SIRT1 function is its link with the Wnt signalling pathway. It is well established that Wnt signalling orchestrates many of the same diverse processes as SIRT1. Wnt ligands transmit signals through specific Frizzled (FZD) or FZD/LRP5/6 co-receptor complexes [17]. These signals are transmitted through Dishevelled (Dvl) proteins that direct canonical (β-catenin-dependent) or non-canonical (β-catenin-independent) signalling [18]. Most of the mechanistic insights into Wnt signalling have been downstream of the FZD receptors, and studies identifying regulators of FZD expression have been lacking. Early studies have shown that blocking FZDs could inhibit angiogenesis and tumor growth [19], and application of the purified extracellular domain of FZD7 could decrease β-catenin/TCF4 transcriptional activity [20]. More recently, one study reported that use of an anti-FZD antibody inhibited the binding of Wnts to FZD (such as FZD7) and inhibited the growth of human tumor xenografts [21]. Here, we describe for the first time an important functional link between SIRT1 and FZD7, which has recently been implicated in breast cancer pathogenesis [3]. We report that SIRT1/2 positively regulates FZD7 mRNA and protein levels. Additionally, we show that β-catenin and c-Jun occupy the promoter region of the FZD7 gene, in a SIRT1/2 dependent manner. We have uncovered a new mechanism for the regulation of FZD7 and provide a critical new link between the sirtuins and FZD7, one of the earliest nodal points from which oncogenic Wnt signaling emanates. This study demonstrates that inhibition of specific sirtuins may provide a unique strategy for inhibiting the constitutively active Wnt pathway in cancer cells at the level of the receptor.

## Results

### Frizzled 7 expression in breast cancer cells

**Figure 1:**
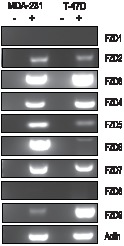
Variable frizzled receptor expression in breast cancer cell lines. **A**. Semi-quantitative RT-PCR analysis of frizzled receptors 1–9 to determine expression pattern in MDA-MB-231 cells and T-47D cells. Samples were resolved on 1% low melting agarose gel.

The earliest evidence that the Wnt pathway may be subject to regulation by SIRT1 came from studies demonstrating that SIRT1 localizes to the promoter of the gene encoding Wnt antagonist, *secreted frizzled related protein 1* (SFRP1) and directly contributes to its aberrant epigenetic silencing [9]. This work demonstrated a connection between SIRT1 and Wnt antagonists. Subsequent studies extended this connection between the sirtuins and the Wnt pathway by demonstrating that SIRT1 was involved in stabilizing the levels of all three Dvl proteins [11]. Because SIRT1 did not significantly regulate Dvl at the level of transcription, we searched upstream of Dvl at the level of the receptor to see if the sirtuins regulated Wnt signalling via the Frizzled receptors. To address this question, we systematically analyzed the impact of loss of SIRT1 function on several members of the Frizzled (FZD) family of receptors in two cancer cell lines previously shown to be sensitive to SIRT1 inhibition, MDA-MB-231 and T-47D cells. First, we determined the expression pattern of FZD genes, as previous studies suggested that autocrine Wnt signalling may be important for cancer cell viability. We performed RT-PCR analysis and observed a variable pattern of expression (Fig 1). FZD1 and FZD8 levels were low to undetectable in both cell lines, while FZD3, FZD4 and FZD7 were strongly positive across both cell lines. Because FZD7 was expressed in both cells lines, been previously implicated in breast cancer malignancy [3], [22], and appeared to be a target in pilot studies, we focused on this FZD member exclusively in this study.

### Sirtuin1/2 inhibition decreases expression of FZD7 mRNA

**Figure 2:**
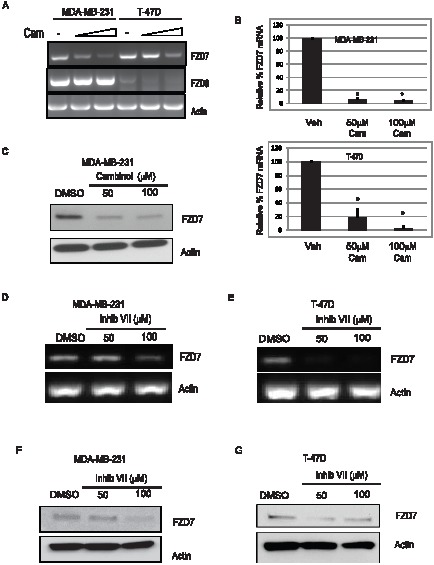
Pharmacologic inhibition of SIRT1/2 decreases FZD7 mRNA expression in both MDA-MB-231 and T-47D cells. **A**. MDA-MB-231 and T-47D cells were treated with 50 and 100 µM cambinol for 24 hrs. RNA was isolated from cells for cDNA synthesis. RT-PCR was performed to quantitate gene expression changes. **B**. MDA-MB-231 (upper) cells and T-47D (lower) cells were treated with 50 and 100 µM cambinol for 24 hrs. Cells were harvested and RNA isolated for analysis of mRNA via quantitative real-time PCR. **C**. MDA-MB-231 cells were treated with cambinol for 24 hrs. Following drug application, cells were lysed in RIPA buffer for protein analysis. **D**. and **E**. RNA was harvested from MDA-MB-231 or T-47D cells (respectively) that had been treated for 18 hrs with inhibitor VII, and analyzed using semi-quantitative PCR techniques. **F**. MDA-MB-231 cells were treated with inhibitor VII for 18 hrs, and then lysed in RIPA buffer for protein analysis. **G**. T-47D cells were treated the same as MDA-MB-231 cells with inhibitor VII for 18 hrs, and then protein was analyzed via Western blot. Data plotted are from average of 3 experiments +/− S.E.M, * p-value <0.05.

To determine whether the sirtuins regulated FZD7 expression, we treated MDA-MB-231 and T-47D cells with cambinol, an inhibitor of SIRT1 and SIRT2 [23]. Following treatments, we observed a reduction in the levels of FZD7 mRNA in both MDA-MB-231 and T-47D cells (Fig 2A – top panel). While we did notice that inhibition of SIRT1/2 led to decreased FZD7 mRNA, we did not observe any significant change in FZD6 in MDA-MB-231 cells (Fig 2A – middle panel). The levels of FZD6 were too low to assess changes in T-47D cells following cambinol treatment (Fig 2A – middle panel). To determine the relative change in FZD7 gene, we performed real-time PCR analyses (RT-qPCR) in both breast cancer cell lines following treatment with either 50 µM or 100 µM of cambinol. Our results were consistent with the semi-quantitative analysis, in finding a substantial decrease of nearly 90% with 50 µM cambinol in MDA-MB-231 (Fig 2B Upper) and 80% with same dosage in T-47D cells (Fig 2B Lower). This result led us to ask whether inhibition of SIRT1/2 activity might also lead to changes in protein levels.

### Sirtuin inhibition decreases expression of Fzd7 protein in vitro

Once it was evident that SIRT1/2 activity had influence on FZD7 mRNA expression, we next determined whether the decrease in FZD7 mRNA resulting from cambinol treatments corresponded to decreases in FZD7 protein. Protein analysis of cells treated with increasing doses of cambinol showed a reduction in FZD7 protein, similar to that observed during PCR studies (Fig 2C). To extend our analysis of FZD7, we also incorporated an additional small molecule inhibitor of SIRT1/2, “inhibitor VII” to our study. MDA-MB-231 and T-47D cells were treated with VII in a similar fashion to the cambinol treatments. Previous studies demonstrated that cambinol specifically inhibits SIRT-1 and SIRT-2, but none of the other sirtuins, nor class I/II histone deacetylases (HDACs) [23]. Inhibitor VII, based on computer-aided molecular structure analysis, is predicted to dock onto the acetyl lysine substrate channel of SIRT-1 and SIRT-2, and have little interaction with other sirtuins. Using RT-PCR, we were able to demonstrate a decrease in FZD7 mRNA in 231 and T-47D cells following inhibitor VII treatment, analgous to cambinol treatment (Fig 2D and 2F). Inhibitor VII also proved to be effective at decreasing FZD7 protein levels in both 231 and T-47D cells, particularly at the higher concentrations (Fig 2F and 2G).

**Figure 3:**
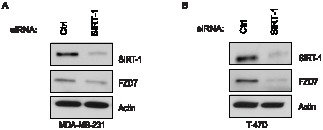
SIRT1 expression is positively correlated with FZD7 protein expression in vitro. Cells were treated with siRNA to reduce the levels of SIRT1 and Western blot analysis of siRNA-mediated knockdown of SIRT1 in MDA-MB-231 (**A**) or T-47D (**B**) cells following 48 hr transfection protocol, using oligofectamine.

In order to confirm the specificity of our pharmacological experiments, we transfected cells with siRNAs against SIRT1. Following transfections, levels of SIRT-1 knock-down were sufficient to recapitulate SIRT1/2 loss of function, showing a decrease in FZD7 protein in both 231 (Fig 3A) and T-47D cells (Fig 3B). These data are the first evidence, to our knowledge, that demonstrated that SIRT1/2 inhibition perturbs FZD7 mRNA and protein levels, suggesting that sirtuins may play a regulatory role in FZD receptor expression.

### β-catenin and c-Jun is enriched at Fzd7 promoter of breast cancer cells

**Figure 4:**
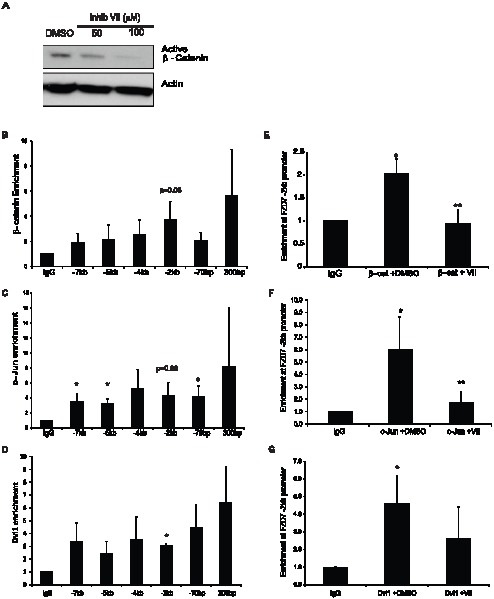
β-catenin regulation of FZD7 is sirtuin-dependent in breast cancer cells. **A**. Cells were treated with 50 and 100 µM of inhibitor VII, and protein was harvested in RIPA buffer to analyze active b-catenin expression. **B**. Chromatin immunoprecipitation (ChIP) for β-catenin at the promoter of FZD7 gene in T-47D cells, followed by qPCR with primers that target the domains through -7 kb region of FZD7 promoter/enhancer (Table1). **C**. ChIP analysis of c-Jun enrichment at the FZD7 promoter/enhancer. T-47D cells treated with inhibitor VII or vehicle for 24 hrs and then ChIP performed as previously described. **D**. ChIP analysis of Dvl1 enrichment at the FZD7 promoter/enhancer. **E, F and G**. T-47D cells were treated with 100 µM of inhibitor VII for 18 hrs. Cells were then harvested for ChIP-qPCR analysis. Data plotted are from average or 4 experiments +/− S.E.M. An * indicate a p-value <0.05 versus IgG sample, A ** indicates a p-value <0.05 versus DMSO treated samples. Samples were normalized to non-immune IgG, (n = 4).

**Figure 5:**
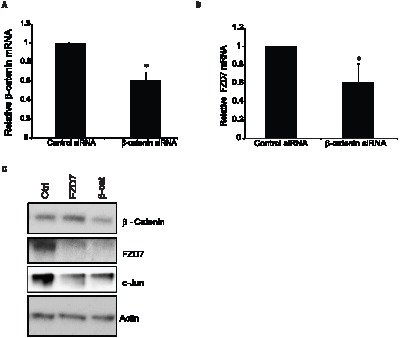
β-catenin knockdown recapitulates the affect of SIRT1/2 inhibition on FZD7. T-47D cells were treated for 24 hrs with siRNA against β-catenin (**A**) or FZD7 (**B**). Expression of related genes (β-catenin, FZD7, and GAPDH) were measured by real-time PCR. Gene expression was normalized to GAPDH. **B**. T-47D cells were transfected with siRNA against either β-catenin or FZD7 for 48-hours using oligofectamine, cells were processed for Western blot analysis probing for β-catenin and FZD7. An * indicates a p-value <0.05 versus control. Samples were normalized to non-immune IgG, (n = 3).

Although the potential of sirtuins functioning as transcriptional regulators of FZD7 was high, there were still gaps in our understanding of how sirtuins had such an effect on the FZD7 receptor. We reasoned that if SIRT1/2 were regulating FZD7 transcription, then perhaps it affected the action of proteins at the FZD7 gene promoter. Based on data available from NCBI, the FZD7 gene is located within human genome sequence AC069148.6. It had been previously reported that there are several conserved transcription factor binding motifs present in the promoter region of FZD7 including; PU.1, SP1, CCAAT-box, and TCF/LEF sites [24]. Therefore, we began to investigate which factors were present at promoter of FZD7 in breast cancer cells under basal conditions. Previously, our laboratory demonstrated that SIRT1 activity has the ability to regulate the expression of Wnt target genes, and was able to reduce the level of active β-catenin protein in T-47D cells (Fig 4A) [11]. Another study had also shown that FZD7 expression was regulated by β-catenin in colorectal cancer cells [25]. So to test whether β-catenin was involved in FZD7 expression in breast cancer cells, we performed chromatin immunoprecipitation (ChIP) followed by quantitative PCR (ChIP-qPCR). We found that β-catenin appeared at varying levels across the entire 7 kb region upstream of the transcription start site that was examined (Fig 4B). Interestingly, it has been previously shown that β-catenin forms complexes with transcription factors such as c-Jun and Dvl1, 2, and 3 to mediate its affects on transcription [26]. Therefore, we conducted ChIP-qPCR analysis for c-Jun and Dvl1 at the FZD7 promoter as well, and found their enrichment patterns closely resembled that of β-catenin, suggesting that they may be co-localized together (Fig 4C and 4D). To determine if SIRT1/2 activity affected β-catenin, c-Jun, or Dvl1, we performed ChIP-qPCR on cells that had been treated with 100 µM inhibitor VII for 18 hrs. Our data revealed that β-catenin and c-Jun were sensitive to SIRT1/2 inhibition to varying degrees across the entire 7 kb promoter/enhancer region; however we narrowed the focus to -2 kb upstream of the TSS, where the level of β-catenin enrichment was most consistent (Fig 4B). In the presence of drug, we saw significantly less β-catenin and c-Jun at the -2 kb region of FZD7 promoter (Fig 4E and 4F). However, Dvl1 did not appear to leave the -2 kb promoter region after treatment to the same extent, indicating that it may not be regulated in the same fashion as β-catenin or c-Jun (Fig 4G). From these data, we proposed that any perturbations in β-catenin or c-Jun expression would in turn, affect expression of FZD7. To explore this idea further, T-47D cells were treated with inhibitor VII for 18 hrs and protein was harvested in order to determine levels of β-catenin, c-Jun, and Dvl1 following our treatment regimen. Inhibitor treated cells showed a decrease in c-Jun at both concentrations of inhibitor utilized, while Dvl1 levels were not changed (data not shown). To confirm the inhibitor studies, we took T-47D cells and transfected them with siRNA against either β-catenin or FZD7. Following transfections, RNA and protein were isolated for analysis and the resulting level of β-catenin knockdown was assessed using qPCR analysis (Fig 5A). Additionally, knockdown of β-catenin correlated with decreased FZD7 gene expression (Fig 5B) and a sharp decrease in protein, when compared to the knockdown of FZD7 in the preceding lane (Fig 5C). We also observed a drop in the level of c-Jun, as we had seen with SIRT1/2 inhibitor treatments; however the exact reason for this effect was unknown, but was likely connected to deregulation of the Wnt signalling pathway. These data as well as our previous studies suggested that β-catenin and c-Jun are integral to the relationship between SIRT1 activities and FZD7 expression.

### Fzd7 knockdown inhibits cell migration and proliferation in breast cancer cells

**Figure 6:**
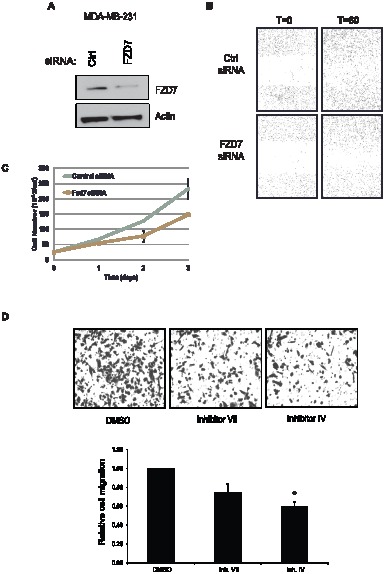
SIRT1/2 activity and FZD7 expression are critical to cell motility and growth. **A**. MDA-MB-231 cells were transfected for 24 hrs with siRNA against FZD7 and protein expression of FZD7 was later determined by Western blot analysis. **B**. MDA-MB-231 cells that had been transfected with FZD7-specific siRNA 24 hrs were placed on 96-well plates for wound healing/migration assay. Wound healing was performed for total 72 hrs to allow complete wound closure. **C**. Following 24 hrs transfection of MDA-MB-231 cells with FZD7-specific siRNA, cells were plated in triplicate to measure growth rates over 72 hrs. Each point plotted is the average of 3 independent experiments. **D**. MDA-MB-231 cells were treated with SIRT1/2 inhibitor VII at 50 µM, and placed on transwell plates for migration analysis. Migration was conducted for 14 hrs. Transwells were then washed and stained with crystal violet before visualization on microscope. Bar graph depicts 3 experimental replicates and error bar is the S.E.M. An * indicates a p-value <0.05 versus control.

Once we had established some link between, β-catenin, c-Jun, and the FZD7 gene, we wanted to determine the physiological consequence of altered FZD7 expression in breast cancer cells. Ueno and colleagues demonstrated that FZD7 expression is important for cell survival in colorectal cancer; however the role that SIRT1 plays had not been explored in breast cancer cells. We decided to test whether cell motility was influenced in cells with reduced FZD7 levels by utilizing MDA-MB-231 cells, due to their high level of motility as compared to T-47D cells. We knocked down FZD7 using siRNA as previously described and confirmed FZD7 expression (Fig 6A). Knockdown of FZD7 protein led to inhibition of cell motility as determined by wound healing assay, (Fig 6B). These data were in agreement with previous studies done by Yang and colleagues, who had determined that cell growth and invasiveness were blunted in FZD7 shRNA transfected triple-negative breast cancer cells [3]. To further validate the congruity between our data and published findings, we measured cell growth over time to determine whether a decrease in proliferation in FZD7 siRNA transfected cells. FZD7 depletion did in fact correlate with a noticeable lag in cell growth over the 72-hrs of the assay (Fig 6C). The reduction of FZD7 in these cells hindered the ability of the cells to grow and migrate under basal conditions; however, to make the connection clear we needed to test the effect of sirtuin inhibition on cell motility. Cells were treated with 50 µM of inhibitor VII, and plated into transwell dishes for 14 hrs. Following migration, cells were harvested and stained for microscopic analysis. In parallel, cells that had been treated with inhibitor VII were plated for viability assays, in which there were no significant changes in viability over the course of treatment (data not shown). Cell migration was hindered by the addition of inhibitor VII, although the differences did not reach statistical significance (Fig 6D, middle panel). To look more specifically at the effects of SIRT1 inhibition on cell motility, we treated cell with a SIRT1 specific inhibitor, inhibitor IV. We observed a more potent suppression on migration in inhibitor IV treated cells than with inhibitor VII (Fig 6D, lower panel); demonstrating that SIRT1 is a major component of cell motility in these cells. These findings indicate that SIRT1/2 may regulate cell growth and motility by controlling the expression of a major Wnt ligand receptor in breast cancer cells.

## Discussion

Wnt signaling is necessary for proper cell growth and development. However, in tumors, Wnt signaling is often dysregulated through mutations or some other type of hyper-activation event that leads to aberrant cell growth [22], [27]–[30]. Due to this well established connection with tumorigenesis, it is important to better characterize oncogenic Wnt signaling in order to target this pathway to treat disease. The Wnt pathway functions through an autocrine/paracrine- like action, wherein Wnt proteins are secreted from cells so that they may bind to their cognate receptors, the Frizzleds (Frizzled 1–10). The binding of the Wnt ligands to the Frizzled receptors commences a signaling cascade that activates both the transcription of β-catenin-dependent and independent Wnt target genes, which ultimately lead to cell growth and/or cell migration. In the current study, we have shown for the first time that the class III deacetylase, SIRT1, is a positive regulator of the Wnt receptor FZD7.

Previous studies have demonstrated that FZD7 is a critical factor for tumorigenesis. In one study a significant correlation between the expression of FZD7 and *in vivo* tumor formation was shown; indicating that FZD7 is essential for cancer cell development [3]. In our own data, it appears that knockdown of FZD7 suppresses cell motility. Interestingly, Mei and colleagues demonstrated that the expression of FZD7 is concomitant with the undifferentiated state of limbal stem/progenitor cells (LSCs) [31]. These progenitor cells possess many of the characteristics that cancer cells acquire as disease progresses, suggesting FZD7-mediated Wnt signaling may have a role in the transition of normal differentiated cells to more plastic phenotypes. Similarly, the epithelial to mesenchymal transition (EMT) is a classic hallmark of tumor metastasis, which has been shown to be induced by SIRT1 activity in certain cancers [32]. Considering this, it would be interesting to see if future studies reveal a connection between EMT induction via SIRT1-FZD7 signaling. SIRT1 inhibition could possibly be considered a target for treatment of metastatic cancers where both FZD7 and SIRT1 are overexpressed.

Our work shows that SIRT1 positively regulates the activity of β-catenin, which agrees with published work from others, wherein acetylation functions as a molecular switch to control the activation potential of β-catenin [3], [33]–[37]. While other published studies have described the role of β-catenin in the regulation of FZD7 promoter activity in colorectal cancer, our study extends these observations to ER-positive and ER-negative breast cancer cells. This suggests that this connection may be a common feature in cancer cells. When SIRT1/2 is inhibited we observe changes in both β-catenin and c-Jun. Due to evidence that β-catenin, c-Jun, and Dvls can form a complex in the nucleus, we suspect that these factors may be part of a transcriptional complex [35].

Inhibition of SIRT1/2 presents a blockade in both the canonical and non-canonical Wnt-pathway that is mediated through the subsequent down-regulation of FZD7 receptor. Interestingly, Wang and colleagues have demonstrated an antagonistic relationship between the activation of the Ca^2+^-Wnt pathway and the β-catenin-dependent canonical pathway [38]. Therefore targeting FZD7 expression would potentially affect cellular growth via the canonical pathway and cell movement via planar cell polarity and Ca^2+^- associated Wnt pathways without the potential feedback mechanisms engaging. This is consistent with our results with both FZD7 specific siRNA and SIRT1/2 inhibition where cell growth and motility is stunted in culture. Though beyond the scope of this study, it would be of interest to examine the relationship between FZD7, Dvl, and heterotrimeric G-proteins and investigate their combined contribution to the development and maintenance of cancer cells.

Understanding the mechanisms regulating FZD7 expression in highly invasive cancers is important to the development of more efficacious treatments. The presence of constitutive Wnt signaling is known to drive the formation of cancer in mammary tissues, as such, it is an ideal candidate for targeted therapy [28]. Based on our data and previous studies, we speculate that treatment of cancer cells with sirtuin inhibitors could be an effective method to deprive cancer or pre-cancerous cells the unchecked growth potential that allows for tumor development.

To date there are no sirtuin specific treatment options for cancer patients, though there are many drugs designed to inhibit the enzyme. As we continue to generate data detailing the mechanisms of sirtuin activity in the cell, the significance of sirtuin regulation to improving health outcomes of patients will become more evident. The results from our study agree with published research in that FZD7 has an important role in cancer cell growth and development [35], and we have shown that SIRT1 can regulate the expression of this critical receptor at the transcriptional level. Perhaps by combining current frontline therapies, with inhibitors of SIRT1 or SIRT2, along with a FZD7 antagonist, we can create a synergistic effect in cancers with an over-active Wnt pathway.

## Materials and Methods

### Cell culture

Cell lines were obtained from the American Type Culture Collection. MDA-MB-231 cells were cultured in Dulbecco's Modified Eagle's Medium supplemented with 10% fetal bovine serum and 1% penicillin/streptomycin (Invitrogen, Carlsbad, California, United States). T-47D cells were cultured in 1640 RPMI medium supplemented with 10% fetal bovine serum,0.2 units/ml insulin, 1 mM sodium pyruvate, and 1% penicillin/streptomycin (Invitrogen).

### Endpoint and real time quantative PCR

For endpoint PCR analysis intron-spanning primers specific for human frizzled receptor 1–9 and β-actin were used (Table 1). Total RNA was isolated using Trizol (Invitrogen), and 2 µg of RNA was reverse-transcribed with M-MLV Reverse Transcriptase (Promega). Endpoint PCR reactions utilized JumpStart RedTaq (Sigma) on Veriti thermocycler (Applied biosystems). Real-time PCR Reactions for were ran on CFX96 well Real-Time PCR System (Bio-Rad) and utilized PerfeCTa SYBR green FastMix, ROX (Quanta Bioscience, Inc., MD, cat. number 95073-012).

**Table 1:** List of PCR primers.

Gene	Forward Primer	Reverse Primer
**Expression**		
FZD1	TCATGAATCGCAAGTTTCCGCGG	GGCACAAAGTTCCCAGCTCGC
FZD2	GTGCAGTGCTCGCCCGAACT	GGTGCTCCAGCGTGGCGTAG
FZD3	ACCTCTGACGGTGCGAAGAGT	AGGGTGGAATGGCTCCATTGCC
FZD4	TTGGGCACGAGCTGCAGACG	TGAGCACACAGTTCAGGCTCCT
FZD5	GACCCAGGGACGGAGGACCC	TCCTCGCCGGATAGGGCTGG
FZD6	GGACGGAGCTAGCACCCCCA	ACAGTGCATAGGTCACTTCCAGTGT
FZD7	CGCGGCCGCTCCGCTTTC	GCGCTCGCACAGAGAACGACA
FZD8	TGGACTACAACCGCACCGACCT	AAGACGGTGAAGGCGCGCTC
FZD9	CCCGCGCTCAAGACCATCGTC	AGGCGCCAGAAGTCCATGTTGA
Actin	GGACTTCGAGCAAGAGATGG	AGCACTGTGTTGGCGTACAG
**ChIP primers**		
FZD7–7 kb	ACAAGTTTCCCACTTGCCCT	GACCCCTTGAACATTCCACCT
FZD7–5 kb	AATGATCCAACACCCCAACA	CCCAGTCCTGCGGTTGTAAT
FZD7–4 kb	TGCCTAACATCGGAGCTGTC	TGCTTATTCATCAGGCCACTG
FZD7–2 kb	GAGGCCCCCTGGTGACTTAT	CTCGTTCTCGGGTAGCGTTT
FZD7–70 bp	CCGCATCCAAGCCTCTCC	CGGCCGCGAGTGCAG
FZD7 300 bp	GAGGTGCACCAGTTCTACCC	CGAACTTGTTCATGAGCGCC

### Transient transfections

Transfections were performed with Oligofectamine according to the manufacturer's protocol. Unless stated otherwise, cells were seeded at 2×10^5^ per well in a six-well dish and transfected with 100 nM siRNA for 24 or 48 hours with transfections. ONTarget siRNAs were obtained from Dharmacon for the following targets: random sequence control (D-001810-01-20), SIRT1 (M-003540-01-0005), FZD7 (L-003671-00-0005), and β-catenin (M-003482-00-0005). For inhibitor studies, cambinol (Sigma C0494) and inhibitor VII (Calbiochem 566327) were resuspended in DMSO.

### Western blots

Antibodies and dilutions used in western blots are as follows: Membranes were incubated in 5% milk/TBST with primary antibody overnight at 4°C. Membranes were washed briefly with TBST and probed with horseradish peroxidase (HRP) -conjugated secondary antibodies in 5% milk/TBST for 1 hour at room temperature. Membranes were washed with TBST and deionized water before visualization by enhanced chemiluminescence. Antibodies used in this study were the following: β-catenin (Cell Signaling 9562), Frizzled 7 (Sigma clone 4D9), β-Actin (Santa Cruz C-4), Dishevlled-2 (Cell Signaling D1196), SIRT1 (Epitomics 1104-1).

### Chromatin Immunoprecipitation (ChIP)

T-47D cells were grown to confluence in 10 cm dishes; a final cell count of approximately 15x10^6^ cells/plate. Proteins were cross-linked to DNA using formaldehyde added directly to the culture medium at a final concentration of 1% for 10 minutes at room temperature. The cross-linking reaction was quenched by adding glycine to a final concentration of 0.125 M for 5 minutes at room temperature. The medium was then removed and cells were washed with 1× PBS containing a protease inhibitor cocktail. Then cells were scraped, pelleted and washed twice with PBS plus protease inhibitor cocktail as described above. Cells were resuspended in SDS Lysis Buffer (Millipore 20-163) plus protease inhibitor cocktail. Cells were sonicated in a Diagenode Bioruptor sonicator for 30 cycles of sonication (30 second pulses and 30 second rest). The soluble chromatin fraction was quantitated and 100 mg of chromatin was precleared with 10 ul of Protein A Dynabeads (Invitrogen) for 2 hours, then incubated overnight at 4°C with either β-catenin (Cell Signaling; 9562), c-Jun (Santa-Cruz, H-79), Dvl1 (Sigma, 3570), or rabbit IgG (Sigma I5006). Dynabeads were added to the chromatin-antibody mixture and incubated with rotation for 2 hour at 4°C. ChIPs were washed with a low salt wash buffer (Millipore 20–154), high salt wash buffer (Millipore 20–155), and TE (Millipore 20–157). Crosslinks were reversed overnight at 65°C, followed by RNAse (Promega) at 37°C for 2 hour, and proteinase K (Promega) at 55°C for 2 hours. DNA was eluted using Qiaquick PCR purification kit (Qiagen) and amplified by PCR.

### Wound healing

Transfected MDA-MB-231 cells (5×105) were suspended in serum-free DMEM media, and from this suspension 100 µl was placed into 96-well plate in quadruplicate. The cells were allowed to adhere overnight. Cells were scratched with automatic wound maker from INLET core lab. Cells were washed with twice with PBS and complete media was added to cells. Plates were placed inside INCUCYTE system and migration was monitored over 60 hrs. Images were captured automatically every 4 hrs for duration of experiment.

### Growth curve

Cell Growth Curve was determined by taking transfected and control transfected cells and plating equal cell numbers into 12-well plate. Triplicate wells were counted for each transfection condition for each day, and the average was plotted on a graph. The variation in cell numbers is from multiple experiments not individual wells. Cell viability was measured using Cell Titer Blue Viability Assay obtained from Promega (G8080) and performed according to the manufacturer's instructions. Fluorescence was measured using a BioTek Synergy HT.

### Statistical analysis

All experiments were reproduced three times, and representative experiments are shown. The averages of repeat experiments were plotted and the standard deviation of mean or absolute deviation from mean was calculated from experimental values. The student *t* test was used to determine statistical significance of the differences. An * signifies a p-value of 0.05 or less, a ** indicates comparison to experimental control sample.

### Data availability

Data related to studies described herein are available upon request to interested researchers. Contact corresponding author to make arrangements.
